# Thyroxine Replacement for Subfertile Females With Subclinical Hypothyroidism and Autoimmune Thyroiditis: A Systematic Review

**DOI:** 10.7759/cureus.16872

**Published:** 2021-08-04

**Authors:** Revathi Myneni, Harsh V Chawla, Amit S Grewal, Govinathan Vivekanandan, Andrew Ndakotsu, Ansha P Abubacker, Aimen Iqbal, Safeera Khan

**Affiliations:** 1 Family Medicine, California Institute of Behavioral Neurosciences & Psychology, Fairfield, USA; 2 Internal Medicine, California Institute of Behavioral Neurosciences & Psychology, Fairfield, USA; 3 Department of Research, California Institute of Behavioral Neurosciences & Psychology, Fairfield, USA; 4 Emergency Medicine, California Institute of Behavioral Neurosciences & Psychology, Fairfield, USA

**Keywords:** levothyroxine, thyroxine, subclinical hypothyroidism, autoimmune thyroiditis, subfertile woman, infertility female, hashimoto thyroiditis

## Abstract

The second most prevalent endocrine condition affecting women of reproductive age is thyroid disease. The difference between an increased thyroid-stimulating hormone (TSH) concentration and a normal free thyroxine hormone level is used to identify subclinical hypothyroidism. Thyroid autoantibodies, independent of thyroid hormone levels, are used to diagnose autoimmune thyroid disease (ATD). Thyroxine can help infertile women with these two types of thyroid illnesses have better birth outcomes during fertility treatment. We performed a systematic review using PubMed (Medline) as a major database and some other sources EMBASE, the Cochrane Library, Web of Science, Scopus, and Science Direct. We concentrated on four studies, including 806 patients. Our goal is to investigate the efficacy and risks of levothyroxine therapy in infertile women who are receiving fertility treatments and have subclinical hypothyroidism or adequate thyroid function as well as thyroid autoimmunity (euthyroid autoimmune thyroid disorder). Thyroid activity in hypothyroid women should be tracked at pregnancy confirmation and closely monitored during the pregnancy. Early in pregnancy, the dosage of levothyroxine (LT4) can be raised. To ensure optimum TSH levels during breastfeeding, we recommend that patients who are followed in the primary sector have their LT4 dose increased by their general practitioner before their first referral to an endocrinological outpatient clinic. It's important to pay more attention to and track pregnant women with hypothyroidism, who consider pregnancy, to get the best results. LT4 therapy can help subfertile women with subclinical hypothyroidism who are having in vitro fertilization (IVF)/intracytoplasmic sperm injection (ICSI) since it improves embryo growth, implantation rate, and live birth rate.

## Introduction and background

Thyroid disease is the second most common endocrinopathy among women of reproductive age. When peripheral thyroid hormones are within the standard recommended ranges but serum thyroid-stimulating hormone (TSH) levels are slightly high, subclinical hypothyroidism, also known as mild thyroid insufficiency, is diagnosed. Thyroid peroxidase antibodies (TPOAb) are used to diagnose autoimmune thyroid illness. TPOAb are antibodies to an enzyme located in the thyroid gland that plays a key part in the production of thyroid hormones [1-3]. 

A high blood thyrotropin level combined with a typical free thyroxine (fT4) level is used to make a scientific diagnosis of subclinical hypothyroidism (SCH). Existing guidelines indicate a maximum serum TSH level of 2.5 mIU/L for the first trimester and 3.0 mIU/L for the second and third trimesters of pregnancy [1]. The correlation of SCH with an elevated risk of one or more undesirable pregnancy effects, most notably miscarriage, preterm labor, gestational hypertension, and low birth weight, has been observed in many retrospective trials comparing euthyroid pregnant women with those with uncontrolled SCH [2,4]. 

There are different effects of thyroid hormones on ovarian function. They may be required for the development of oocytes and the growth of ovarian follicles. Thyroid hormone receptors were detected in human oocytes, where they work in conjunction with luteinizing hormone and human chorionic gonadotrophin (hCG) receptors to improve granulosa cell function (i.e., progesterone production) and trophoblastic differentiation. [5,6]. 

To boost pregnancy outcomes during assisted reproduction, thyroxine can be a useful medication for subfertile women with these two particular forms of thyroid disease. Hypothyroidism that goes untreated has been related to an increased risk of miscarriage. The objective was to determine the efficacy of raising the dose of levothyroxine (LT4) to reduce the risk of adverse effects for pregnant women with TSH levels above the recommended first-trimester limit [7]. 

It has repeatedly been shown that overt hypothyroidism in pregnancy raises the risk of maternal and fetal abnormalities [8]. While less common than overt, subclinical hypothyroidism during pregnancy also has, but not frequently, been associated with adverse events in some studies [4]. Previous studies have shown that up to 68 percent of pregnant women on LT4 have an elevated TSH level at their first prenatal appointment. A recent large-scale analysis showed that most women treated with LT4 had early gestational TSH levels above the prescribed targets, with an elevated risk of miscarriage [9]. To improve early gestation thyroid function, it has been recommended that pregnant women increase their LT4 dose empirically by 30% on pregnancy [1]. 

Our review question is “After in vitro fertilization treatment (an egg is mixed with sperm outside the body during a fertility procedure) or intracytoplasmic sperm injection (a fertility procedure in which a single sperm is injected directly into an egg), does levothyroxine hormone ingestion improve fertility outcomes for women with autoimmune thyroid disease or autoimmune thyroiditis?” The goal of this study was to offer evidence-based information to fertility practitioners and consumers on the efficiency of thyroxine replacement in improving clinical pregnancy and fertility rates in subfertile women with subclinical hypothyroidism or euthyroid ATD during assisted reproductive technology (ART). 

Why is it important we should perform this review? Through their effects on sex-hormone binding globulin, prolactin, GnRH (Gonadotropin-releasing hormone) production, and coagulation factors, thyroid hormones influence menstrual cycles, both directly and indirectly. The significance of thyroid hormones in oocyte physiology is supported by the fact that serum TSH concentrations are an important indicator of fertilization failure in women undergoing in vitro fertilization (IVF). TSH levels were found to be considerably greater in women who had oocytes that did not fertilize, according to Cramer and colleagues' prospective research [3]. In this study, elevated TSH levels were closely connected to the likelihood of having fewer than 50% of their eggs fertilized for women who had a minimum of one oocyte inseminated. As a result, TSH was shown to be a predictor of low IVF fertility rates, demonstrating the role of thyroid hormones in oocyte physiology [3]. 

## Review

Our study aimed at evaluating the effects of levothyroxine on subfertile women with subclinical hypothyroidism and autoimmune thyroiditis, mainly how doses are adjusted throughout the pregnancy to decrease the chances of miscarriage and other complications. This systematic review can provide insights to the family physicians and endocrinologists in better managing the patients

Materials & Methods 

Eligibility Criteria 

Papers written in English and published after 1996 and available as full-text articles were collected and examined. Adults aged 18 to 41 were included in the study; ART (assisted reproductive technology) treated infertile couples' pregnancy results were measured between women treated with levothyroxine and women treated with placebo or untreated. Participants who had IVF or intracytoplasmic sperm injection for infertility after being diagnosed with subclinical hypothyroidism, euthyroid autoimmune thyroid disease, or both were considered. 

Women aged less than 18 and more than 41, women who had thyroid surgery or radioiodine therapy were omitted from the study. Women having intrauterine insemination (IUI) or natural conception, whether stimulated or unstimulated, women who took medications that impaired the absorption, binding, or metabolism of levothyroxine medication were excluded from the study. 

Information Sources 

We looked for research that dealt with the use of levothyroxine during pregnancy. We included studies when subclinical hypothyroidism or thyroiditis was diagnosed during pregnancy. The entire research is carried out following the PRISMA (Preferred Reporting Items for Systematic Review and Meta-Analyses) 2020 Guidelines. Our research included randomized clinical trials, systematic analyses of randomized and non-randomized control trials, and retrospective studies conducted in journals until May 2021. A comprehensive search was conducted using PubMed (Medline) as the primary index and EMBASE, the Cochrane Library, Web of Science, Scopus, and Science Direct were used as secondary sources. 

Search Strategy 

Using a Boolean AND OR search technique, the following Mesh approach was built for PubMed search. ( "Thyroxine/administration and dosage"[Majr] OR "Thyroxine/adverse effects"[Majr] OR "Thyroxine/drug effects"[Majr] OR "Thyroxine/immunology"[Majr] OR "Thyroxine/pathogenicity"[Majr] OR "Thyroxine/poisoning"[Majr] OR "Thyroxine/toxicity"[Majr] ) OR ( "Thyroxine/administration and dosage"[Mesh:NoExp] OR "Thyroxine/adverse effects"[Mesh:NoExp] OR "Thyroxine/immunology"[Mesh:NoExp] OR "Thyroxine/poisoning"[Mesh:NoExp] OR "Thyroxine/toxicity"[Mesh:NoExp] ) AND ( "Hypothyroidism/diagnosis"[Mesh] OR "Hypothyroidism/diagnostic imaging"[Mesh] OR "Hypothyroidism/drug therapy"[Mesh] OR "Hypothyroidism/etiology"[Mesh] OR "Hypothyroidism/genetics"[Mesh] OR "Hypothyroidism/immunology"[Mesh] OR "Hypothyroidism/pathology"[Mesh] OR "Hypothyroidism/physiopathology"[Mesh] ) OR "Hypothyroidism"[Mesh:NoExp] AND ( "Thyroiditis, Autoimmune/diagnosis"[Mesh] OR "Thyroiditis, Autoimmune/drug therapy"[Mesh] OR "Thyroiditis, Autoimmune/genetics"[Mesh] OR "Thyroiditis, Autoimmune/immunology"[Mesh] OR "Thyroiditis, Autoimmune/pathology"[Mesh] OR "Thyroiditis, Autoimmune/physiopathology"[Mesh] ) OR "Thyroiditis, Autoimmune"[Mesh:NoExp] 

We also used keyword search for EMBASE, the Cochrane Library, Scopus, Web of Science, and Science Direct. The keywords are as follows: Levothyroxine OR Levoxine OR Unithyroid or levoxyl OR synthroid AND Autoimmune thyroiditis OR AND Hashimoto thyroiditis OR Lymphomatous Thyroiditis AND Hypothyroidism OR Underactive thyroid OR low thyroid AND Infertility female OR subfertile woman OR sterile woman AND Female Infertility OR Sterility, Postpartum OR Postpartum Sterility OR Subfertility, Female OR Female Sterility AND Hypothyroidism OR Primary Hypothyroidism OR Thyroid-Stimulating Hormone Deficiency AND thyroxine OR Levothyroxine OR Synthroid OR Levoxine OR Unithyroid or levoxyl AND Autoimmune thyroiditis OR Hashimoto thyroiditis OR Lymphomatous Thyroiditis OR autoimmune thyroiditis AND Female Infertility OR Sterility, Postpartum OR Postpartum Sterility OR Subfertility, Female OR Female Sterility. 

Data Collection Process 

To evaluate the studies that needed further evaluation, two review writers analysed quantitatively the studies that needed further evaluation. As the evidence revealed that hypothyroidism in subfertile women was an issue, we collected the full texts. We obtained the complete article for clarity whether there were any questions on these requirements after scanning the titles and abstracts. Where applicable, a third review author settled differences by dialogue. 

Study Risk of Bias Assessment 

The following methods were used to determine the study's quality: for randomized control trials, the Cochrane risk bias tool was used, the AMSTAR (A Measurement Tool to Assess systematic Reviews) checklist was used for systematic analyses, and the Newcastle-Ottawa tool for retrospective research. Studies that did not offer enough data for analysis were removed. 

Results 

Study Selection 

After an extensive search in PubMed (Med scape), EMBASE, the Cochrane Library, Web of Science, Scopus, and Science Direct, 7258 studies from PubMed and 3654 studies from other sources were included making a total of 10912 articles. Using the endnote basic online version, 2,665 duplicates were discovered and eliminated, resulting in a total of 8247 articles for screening. Two separate authors screened all 8247 publications based on their titles and abstracts. Records excluded by title screening were 4556, records excluded by abstract screening were 3143. A total of 548 full-text articles were assessed for eligibility and 496 full-text articles were excluded with reasons as mentioned in the PRISMA chart. Fifty-two potential articles were again evaluated for quality appraisal and 19 articles were removed due to lack of sufficient data. After screening 33 full-text articles dealt with levothyroxine treatment in subclinical hypothyroidism and autoimmune thyroiditis in pregnant women were finalized. 

Risk of Bias 

There is a bias possibility when it comes to random sequence generation, all four studies included are randomized clinical trials (RCT) and had a low possibility of bias because they used a machine-generated random number series [10-13]. Two studies showed an unclear risk of allocation concealment bias [11,13]. Whereas two other studies showed a low risk of allocation concealment bias [10,12]. All four studies showed a low risk of performance bias, detection bias, attrition bias and unclear risk of bias with selective reporting [10-13]. A summary of the Cochrane risk of bias tool is given in Table 1 below.

**Table 1:** Tabulated summary of Cochrane risk of bias tool

Trait of paper	Abdel Rahman et al. 2010 [10]	Kim et al. 2011 [11]	Negro et al. 2005 [12]	Wang et al. 2017 [13]
Random sequence generation (selection bias)	Low risk	Low risk	Low risk	Low risk
Allocation concealment (selection bias)	Low risk	Unclear risk	Low risk	Unclear risk
Blinding of participants and personnel (performance bias)	Low risk	Low risk	Low risk	Low risk
Blinding of outcome assessment (detection bias)	Low risk	Low risk	Low risk	Low risk
Incomplete outcome data (attrition bias)	Low risk	Low risk	Low risk	Low risk
Selective reporting (reporting bias)	Unclear risk	Unclear risk	Unclear risk	Unclear risk
Other bias	High risk	Low risk	Unclear risk	Low risk

Selection Process 

Two researchers retrieved the preceding types of data from the included papers: first publisher, year of publishing, place, clinical outcome, ART information, causes of subfertility, age, BMI (body mass index), thyroid status reference values, thyroid hormone levels, and treatment options. Data on pregnant women, such as clinical gestation, stillbirth, abortion, and premature delivery, were gathered and represented as numbers of events in both the LT4-treated and control subjects. 

**Figure 1:**
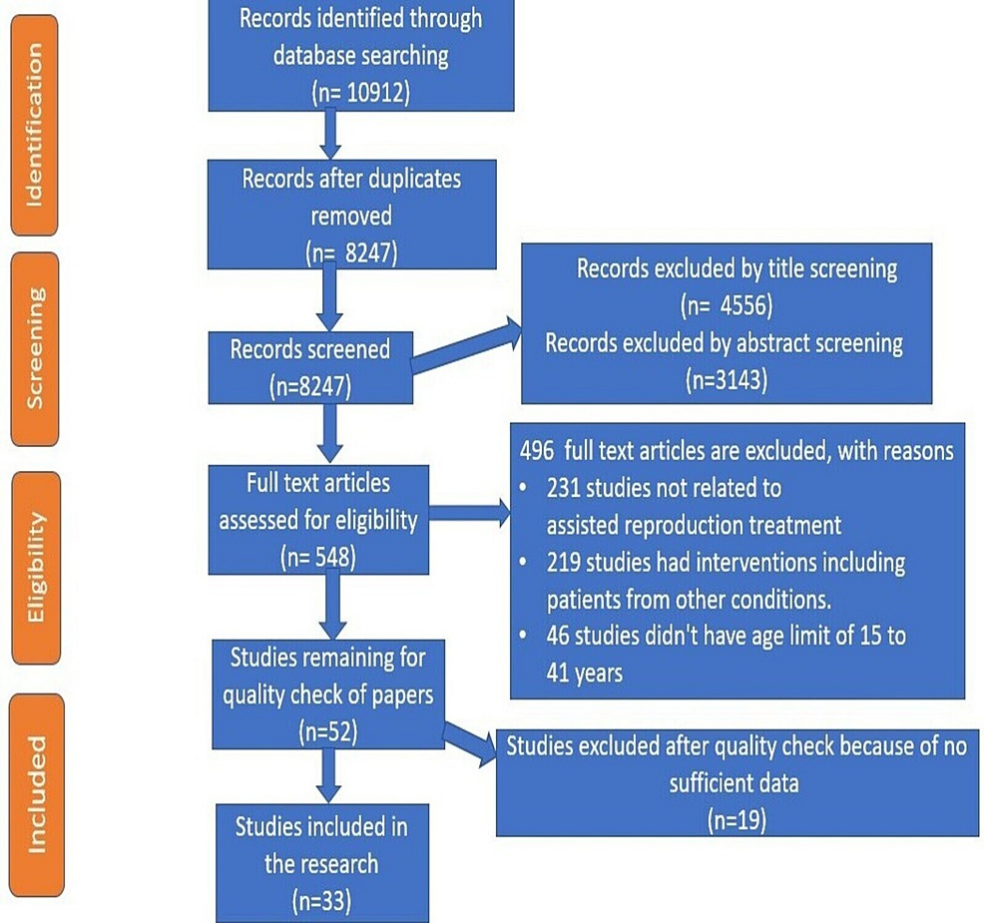
PRISMA Flow Diagram Adapted from source: Page et al. [14] PRISMA - Preferred Reporting Items for Systematic Review and Meta-Analyses

A PRISMA (Preferred Reporting Items for Systematic Review and Meta-Analyses) flow chart documenting the selection process is shown in Figure 1 [14].

The qualitative studies, treatments, and results examined in each clinical trial were all different. The researchers' aims were comparable. Table 2 is a tabular summary of the clinical studies. 

**Table 2:** A tabular summary of the clinical trials RCT - Randomized Clinical Trials, IVF - In Vitro Fertilization, ICSI - Intracytoplasmic Sperm Injection, TSH - Thyroid-Stimulating Hormone,  FT4 - Free Thyroxine, Beta hCG- Beta Human Chorionic Gonadotropin, ART- Assisted Reproductive Technology, BMI- Body Mass Index

Name of Study	Abdel Rahman et al. 2010 [10]	Kim et al. 2011 [11]	Negro et al. 2005 [12]	Wang et al. 2017 [13]
Design	RCT	RCT	RCT	RCT
No. of patients	70	64	72	600
Age (mean)	20-40 years	25-41 years	25-35 years	23-40 years
Inclusion criteria	Aged 20–40 years, daily menstrual periods (21–35 days), and usual thyroid ultrasonography results with thyroid volume of 18 mL and no morphological lesions, minor thyroid failure was the inclusion requirement (subclinical hypothyroidism).	Subfertility mothers who had undergone IVF/ICSI and had subclinical hypothyroidism.	Subfertile woman, Thyroid peroxidase positive antibodies, Patients were undergoing ART treatment.	BMI
Exclusion criteria	Thyroid cancer, goitre (thyroid volume > 18 mL), or other morphological thyroid abnormalities, such as nodules or elevated basal prolactin concentration > 25 ng/mL, are also possible causes and are excluded.	Not mentioned	Women with thyroid dysfunction both overt and subclinical.	Thyroid hormone or antithyroid drug users and those who had thyroid surgery or radioiodine therapy were omitted from the study. Women who had two or more spontaneous miscarriages, diagnosed with diabetes, or other endocrinological or metabolic abnormalities were excluded from the study.
No. Of embryos transferred	One to three	One to four	One to three	One to three
Location	Shatby University Hospital for Women in Alexandria, Egypt	Asan Medical Center, Seoul, South Korea	Department of Endocrinology, District Hospital 'Vito Fazzi', Italy	Peking University Third Hospital, Beijing, China
Participants	April 2006 to April 2007 Women were given an oral dosage of 50–100 g of levothyroxine every morning for a month before starting ART. The levothyroxine dosage was determined by trial and error, starting with a low dose of 50 g and eventually increasing to normalize TSH before IVF. During breastfeeding, the same treatment was continued.	March 2006 to September 2009 From the first day of controlled ovarian stimulation until the serum beta hCG assessment, 50 g of levothyroxine was given every morning. TSH and FT4 levels in the blood were also tested simultaneously as serum beta hCG levels. If conception was confirmed, a stable dose of levothyroxine was administered during the pregnancy.	January 1999 to January 2003 Women received 1 mg/kg/day levothyroxine replacement one month before ART and maintained it throughout pregnancy.	September 2012 to June 2016 Replacement of levothyroxine began 2–4 weeks before the regulated ovarian hyperstimulation and lasted until the end of the pregnancy. Starting doses were 50 g/day for women with TSH levels higher than 2.5 mIU/L and 25 g/day for women with TSH levels lower than 2.5 mIU/L. In the first trimester, the levothyroxine dosage was titrated to keep the TSH level between 0.1 and 2.5 mIU/L, 0.2–3.0 mIU/L in the second trimester, and 0.3–3.0 mIU/L in the third trimester.
Interventions	Control: placebo	When overt hypothyroidism was observed during breastfeeding, including in pregnant women in the control group, levothyroxine's appropriate dosage was added.	Control: placebo	Control: no levothyroxine, but rest everything is the same.
Outcomes	Clinical pregnancy, miscarriage, live birth	Clinical pregnancy, miscarriage, live birth	Clinical pregnancy, miscarriage, live birth	Clinical pregnancy, miscarriage, multiple pregnancies, preterm deliveries
Risk of Bias	Present	Present	Present	Present

Discussion 

A common condition is a subclinical hypothyroidism characterized as elevated TSH levels with normal FT4 levels. Its prevalence in infertile women is greater and increases with the age of women. Autoimmune thyroiditis associated with positive autoimmune thyroid antibodies (ATAs) is the most common cause. There is a strong correlation between elevated levels of TSH and positive ATAs, and patients with ATAs have a higher serum TSH level than patients without antibodies. The likelihood of progressing to severe hypothyroidism is significantly higher in patients with ATAs, and the rate of progression to severe hypothyroidism is likewise strongly related to ATAs levels. Subclinical hypothyroidism can also progress to overt hypothyroidism [11-13]. 

Scientific evidence suggests that women with thyroid disorders have multiple menstrual disorders and decreased fertility associated with them, and these abnormalities are improved by restoring the euthyroid condition. Thyroid hormones work together with follicle-stimulating hormones to increase direct stimulatory effects on granulosa cell functions, including morphologic development. As a result, it can be hypothesized that such an effect could improve oocytes' maturation in the population treated with levothyroxine. This could explain the in vivo and in vitro effects observed in the present study. Consequently, higher implantation and conception rate in response to treatment with levothyroxine could be associated with improved cytoplasmic maturation after human chorionic gonadotropin in the restored oocytes [11,12]. 

Thyroxine (T4) and triiodothyronine (T3) development are increased biologically by the thyroid gland during normal pregnancy to sustain sufficient thyroid hormone concentrations throughout pregnancy. Still, this compensation cannot occur in women with hypothyroidism. In healthy women, overall serum T3 and T4 concentrations rise during the first trimester, hitting a peak either late in the second or early in the third trimester [9]. 

Eight studies out of the thirty-three included studies are randomized clinical trials. Most of the review research articles mentioned about four studies, so in this study, we concentrated on these four research papers and also briefly discussed four more studies. These studies were conducted in Egypt (Abdel Rahman et al.), South Korea (Kim et al.), Italy (Negro et al.), and China (Wang et al.). A total of 806 patients of these four clinical trials are evaluated in our systematic review. 

Abdel Rahman et al. conducted this study in Egypt from April 2006 to April 2007 with 70 patients. For eligibility criteria, women aged 20-40 years with a regular menstrual cycle (21-35 days) and usual thyroid ultrasonography results with thyroid volume of 18 mL and no morphological lesions, minor thyroid failure were included. Thyroid cancer, goiter (thyroid volume > 18 mL), or other morphological thyroid abnormalities, such as nodules or elevated basal prolactin concentration > 25 ng/mL, are also possible causes and were excluded [10]. 

In conclusion, 70 women with subclinical hypothyroidism who've not had their autoimmune antibodies evaluated underwent ART, the dosage of thyroxine was independently balanced to reach a TSH level less than 4.0 mIU/L. This suggested that thyroxine replacement had a stronger impact because women had far higher normal TSH levels before beginning ART than in other trials. We did not use their data in our analyses because we were unsure of the published findings [10]. 

Kim et al. in South Korea from March 2006 to September 2009; the study is a randomized clinical trial with 64 patients. For inclusion criteria, they used subfertility mothers who had undergone IVF/ICSI and had subclinical hypothyroidism, but they didn't mention any exclusion criteria [11]. 

Thyroid antibodies were also tested on 64 women with hypothyroidism who were in the early stages of the disease. In the no-treatment group, thyroid antibodies were higher in the miscarriage subgroup than in the delivery subgroup; whereas, thyroid antibody levels in the treated group were similar. Thyroxine replacement was administered to the patient group at a dosage of 50 g and then titrated to reach a TSH level of less than 2.5 mIU/L. Women in the test group with hypothyroidism after pregnancy were also given a thyroxine substitute, which may have distorted the findings. This study did not offer subgroup results for women with subclinical hypothyroidism who were thyroid antibody positive or negative in the treatment and control groups [11]. 

In conclusion, in infertile women with subclinical hypothyroidism undergoing IVF/ICSI, LT4 treatment will improve embryo quality, implantation rate, and live birth rate [10,11]. An increased risk of miscarriage is associated with both autoantibodies, thyroid peroxidase antibodies (TPOAb) and thyroglobulin antibodies (TGAb) levels, and this negative effect can be reversed by LT4 therapy. For subclinical hypothyroid patients planning IVF/ICSI, LT4 therapy should therefore be considered, and pregnant patients after IVF/ICSI should be treated with an appropriate dose of LT4 during the entire pregnancy period [11]. 

Negro et al. in Italy from January 1999 to January 2003; the study is a randomized clinical trial with 72 patients. For inclusion criteria, they used subfertile women, thyroid peroxidase positive antibodies, patients undergoing ART treatment. Women with overt thyroid dysfunction were excluded. In this prospective trial, we reported no correlation in pregnancy rates among women with and without thyroid autoantibodies. TPOAb (+) women had a higher miscarriage risk than TPOAb (-) women, but TPOAb (+) women treated with LT4 had little difference in miscarriage rate as compared to TPOAb (-). Their findings indicated that when investigating female infertility, the role of autoimmune thyroid disease should be taken into account [12]. 

Nevertheless, since the incidence of TPOAb in their sample group was similar to that of the general population, they conclude that thyroid autoimmunity could not be a cause of infertility in and of itself because the prevalence may have been higher otherwise [12]. 

Wang et al. in China from September 2012 to June 2016; the study is a randomized clinical trial with 600 patients. For inclusion criteria, they included women with Body Mass Index (BMI) < 35 kg/m2 and women undergoing ART treatment. For exclusion criteria, women with thyroid hormone or antithyroid drug users and those who had thyroid surgery or radiotherapy were excluded. Women who had two or more spontaneous abortions, diabetes, or other endocrinological or metabolic abnormalities were also omitted from the study. [13]. 

Both the above studies included 672 women with normal levels of thyroid activity and thyroid autoimmunity [12,13]. They showed that levothyroxine and no therapy resulted in an equal live birth, hospital pregnancy incidence, abortion rate, and multiple or preterm birth rates [12,13]. In Wang et al. study, levothyroxine dosage (25 g or 50 g) was titrated to achieve a TSH level of between 0.1 and 2.5 mIU/L in the first trimester, 0.2 and 3.0 mIU/L in the second trimester, and 0.3 mIU/L and 3.0 mIU/L in the third trimester [13]. Regardless of TSH level, a constant 1 mg/kg/day dose was given to all the groups in this study [12]. 

In order to acquire clinical pregnancies, women with subclinical hypothyroidism who are undergoing in vitro fertilization-intracytoplasmic sperm injection should be given levothyroxine treatment [10]. A longitudinal research in China screened women for thyroid dysfunction during the first trimester of pregnancy and found a correlation between subclinical hypothyroidism and pregnancy failure, but did not indicate any treatment benefits levothyroxine [11]. One major randomized trial aimed to diagnose thyroid dysfunction, which offers indirect proof of the efficacy of LT4 therapy in reducing adverse effects of pregnancy. Hypothyroid pregnant women with serum TSH >2.5 mIU/L and positive levels of TPO antibodies were initiated with LT4. This design research permitted the inclusion of pregnant women with overt hypothyroidism, who are at greater risk for adverse pregnancy and neonatal results, provided that there was no upper TSH cutoff limit and the standard fT4 level was not an inclusion criterion. According to the findings, a significant number of hypothyroid women had at least one negative obstetric or neonatal outcome [12]. Treatment with levothyroxine, relative to no levothyroxine treatment, did not decrease miscarriage rates or raise live-birth rates among Chinese women who had intact thyroid activity and tested positive for anti-thyroperoxidase antibodies and were undergoing IVF-ET (In vitro fertilization & embryo transfer) [13]. 

Some other studies were also briefly mentioned here, Nazarpour and co-authors found that levothyroxine replacement therapy increases birth outcomes and reduces preterm labor and neonatal admissions in TPOAb (+) pregnant women with average FT4. Still, this benefit is only seen in women with TSH >4 IU/mL. Larger randomized controlled trials with appropriate sample sizes and separate iodine and TSH statuses are urgently required to determine if the effects of LT4 therapy in TPOAb (+) women are based on TSH concentrations at treatment initiation [15]. The most well-controlled hypothyroid pregnant women needed increased thyroid hormone dosage after delivery. The dosage of levothyroxine was increased by 50% in the first trimester and then had to be increased by 5% per trimester after that. Since some pregnant women did not need such a change, and in some situations, the levothyroxine dose was also reduced, we suggest that prescription adjustments be made based on test data if they are accessible [16]. Spyridoula and co-authors' study was limited by a small sample size and failed to respond to explanatory variables of pregnancy complications. Single-center research was completed at an academic tertiary treatment center involving primarily white pregnant women with subclinical hypothyroidism. They observed that treatment with levothyroxine was associated with a reduced risk of low birth weight and a low Apgar (appearance, pulse, grimace, activity, and respiration) score in this setting [17]. Rao and co-authors mentioned LT4 supplementation lowers the risk of miscarriage in female patients with thyroid autoimmunity (TAI) and SCH who are receiving IVF/ICSI, according to a recent RCT-based meta-analysis. LT4 therapy, on the other hand, has little effect on the rates of clinical pregnancy, live birth, or preterm birth. More research is required to see whether LT4 intervention will help women with SCH and/or thyroid autoimmunity (TAI) with long-term complications, including neurodevelopmental delays [18]. 

Symptoms, Mood, and Quality of Life 

Subclinical hypothyroidism usually doesn't present with any signs and symptoms. This is particularly valid when the degree of TSH is only slightly elevated. If signs do occur, they are sometimes ambiguous and common, such as nausea, constipation, exhaustion, goiter (swelling in the front of the neck caused by an enlarged thyroid gland), weight gain, hair loss, and cold sensitivity [19]. With the quality of life, thyroid hormone therapy was not statistically associated with changes in the general quality of life. There was a meta-analysis study done on 21 randomized clinical trials, including 2192 patients with subclinical hypothyroidism. The study concluded utilization of thyroid hormone therapy was not associated with changes in the overall quality of life or thyroid-related complications among non-pregnant women with subclinical hypothyroidism. Thyroid hormone treatment may not be used regularly in people with subclinical hypothyroidism, according to these results [20]. 

**Figure 2:**
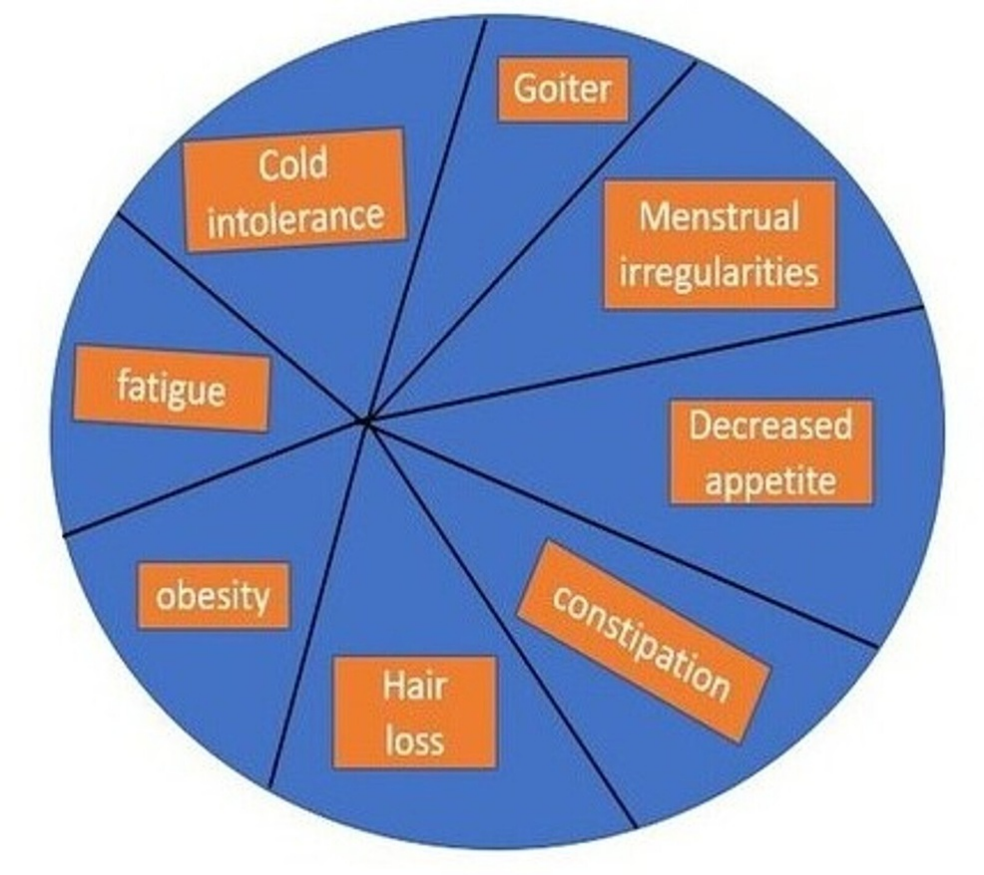
Most common symptoms of hypothyroidism Adapted from source: Villar et al. [19]

In one study, the number of persons experiencing hypothyroidism-related symptoms was marginally but substantially higher than in the euthyroid community [21]. The same findings were not observed in other trials, including patients 85 years and older [22,23]. There were no statistically meaningful changes in seven placebo-controlled blinded trials that looked at these results. In one study, cognitive function increased statistically dramatically [24]. TSH levels were below 15 mlU/L at the start of the research, and the patients had no history of thyroid disease. The Meier review followed the inclusion criteria, but the meta-analysis did not include the subgroup analysis because it applied symptom details only to the treated group [25]. Two studies were included in the meta-analysis assessing health-related quality of life [24,26], and we did not find changes. The most common symptoms of hypothyroidism are shown in Figure 2 [19].

Improvement in Thyroid-Stimulating Hormone (TSH) 

In the three studies we have chosen, we observed some improvement in TSH level when using levothyroxine. In these trials, there was a statistically important difference between the LT4 and placebo categories. In the established group, the total minimum dose necessary to stabilize TSH ranged from 67.5 mcg to 85.5 mcg (range 50 to 125 mcg). TSH levels spontaneously stabilized in 42 percent, 25 percent, and 24 percent of patients in the placebo classes, respectively [24,27,28]. They used patients without thyroid disorder in these trials. Levothyroxine dosage adjustments according to TSH level are mentioned in Table 3 [16].

**Table 3:** Levothyroxine dose adjustment according to TSH level Adapted from source: Kashi et al. [16] TSH - Thyroid-Stimulating Hormone

TSH Level (mIU/L)	Levothyroxine Dosage
TSH≤ 2.5	NONE
TSH≥ 2.5 to 5.0	25 µg increase
TSH≥ 5.0 to 10.0	50 µg increase
TSH≥ 10.0 to 20.0	75 µg increase
TSH> 20.0	100 µg increase

According to some studies there is still a debate regarding the role of thyroxine in influencing miscarriage rates or live births. The new information that this paper provides is when there is an increase in TSH level on lab testing (i.e., TSH>2.5), women of reproductive age (mainly infertile women undergoing fertility treatments) should start taking thyroxine medication to decrease complications during pregnancy and should follow up with their physicians accordingly. It helps with other common symptoms like fatigue, constipation, weight gain, dry skin and other symptoms as these can be masked with pregnancy symptoms but they can be due to decreased thyroid hormone levels. Excess weight gain during pregnancy can cause high blood pressure (which can lead to gestational hypertension, pre-eclampsia, eclampsia, low birth weight and many other complications) and gestational diabetes, which will affect the baby growth and can lead to miscarriages or complications during delivery or decrease live births. To avoid these complications, we should monitor the TSH levels during pregnancy and maintain them in normal levels. 

Future research studies should focus on reproductive age females with ATD (TSH less than 4.0 mIU/L or 2.5 mIU/L), reproductive age females with subclinical hypothyroidism without ATD (TSH more than 4.0 mIU/L or 2.5 mIU/L), and reproductive age females with ATD (TSH greater than 4.0 mIU/L or 2.5 mIU/L). Future trials should report on successful pregnancy and adverse outcomes (including direct thyroxine adverse effects, maternal pregnancy complications, and prenatal complications) as primary outcomes to allow evaluation of the intervention's overall safety and efficacy. Future research is needed to be done to see if LT4 treatments can help women with SCH and/or TAI with long-term complications such as neurodevelopmental difficulties. Future research study should be done with a certain group of people who are undergoing fertility treatment and are diagnosed with ATD or subclinical hypothyroidism should take same brand of levothyroxine medication, monitoring the time of ingestion and not taking any other medications, so it doesn’t impair the absorption of levothyroxine and we should compare the results of how women with SCH or ATD are showing the results. 

Limitations 

The study's drawbacks are largely based on its retrospective existence and the need for certain patients to be removed. We were unable to track certain factors due to the retrospective style of the study. These variables include measuring patients in standard clothing, administering a particular LT4 brand name, monitoring the time of ingestion of LT4, or using a central TSH calculation laboratory. We omitted 75 percent of patients during our chart analysis, mainly because they took medicines that impaired the absorption, binding, or metabolism of LT4. These exclusions increase the data's precision and reliability but also decrease its generalizability. 

## Conclusions

After the extensive analysis of review articles, we concluded that infertile women with subclinical hypothyroidism undergoing IVF/ICSI can benefit from LT4 therapy because it improves embryo production, implantation rate, and live birth rate. An elevated risk of miscarriage is associated with TPOAb and TGAb levels, and this detrimental effect can be reversed by LT4 therapy. The majority of well-controlled hypothyroid pregnant women required a rise in thyroid hormone dosage during pregnancy. The dosage of levothyroxine was increased by 50% in the first trimester and then had to be increased by 5% per trimester after that. Since some pregnant women did not need such a modification, and in some situations, the levothyroxine dose was also lowered, we suggest that prescription changes be made based on test findings if they are accessible. Nevertheless, some studies have not found any increase or decrease in miscarriage rates or live birth rates when using levothyroxine, but these can also be associated by other medical complications, but with most review articles we have studied they concluded that there is improvement with levothyroxine treatment.
